# IgG antibody titers against SARS-CoV-2 nucleocapsid protein correlate with the severity of COVID-19 patients

**DOI:** 10.1186/s12866-021-02401-0

**Published:** 2021-12-18

**Authors:** Li Yang, Qiang Xu, Bin Yang, Jiayu Li, Rong Dong, Jingjing Da, Zhixu Ye, Yongjie Xu, Hourong Zhou, Xiangyan Zhang, Lin Liu, Yan Zha, Fuxun Yu

**Affiliations:** 1 Medical College, Guizhou University, Guiyang, People’s Republic of China; 2 Department of Central Laboratory, Guizhou Provincial People’s Hospital, No. 83 Zhongshan Dong Road, Guiyang, 550002 Guizhou Province People’s Republic of China; 3 NHC Key Laboratory of Pulmonary Immunological Diseases (Guizhou Provincial People’s Hospital), Guiyang, People’s Republic of China

**Keywords:** COVID-19, Recombinant nucleocapsid protein, IgG, ELISA

## Abstract

**Background:**

The 2019 novel coronavirus disease (COVID-19) caused by severe acute respiratory syndrome coronavirus 2 virus (SARS-CoV-2) is a current worldwide threat for which the immunological features after infection need to be investigated. The aim of this study was to establish a highly sensitive and quantitative detection method for SARS-CoV-2 IgG antibody and to compare the antibody reaction difference in patients with different disease severity.

**Results:**

Recombinant SARS-CoV-2 nucleocapsid protein was expressed in *Escherichia coli* and purified to establish an indirect IgG ELISA detection system. The sensitivity of the ELISA was 100% with a specificity of 96.8% and a 98.3% concordance when compared to a colloidal gold kit, in addition, the sensitivity of the ELISA was 100% with a specificity of 98.9% and a 99.4% concordance when compared to a SARS-CoV-2 spike S1 protein IgG antibody ELISA kit. The increased sensitivity resulted in a higher rate of IgG antibody detection for COVID-19 patients. Moreover, the quantitative detection can be conducted with a much higher serum dilution (1:400 vs 1:10, 1:400 vs 1:100). The antibody titers of 88 patients with differing COVID-19 severity at their early convalescence ranged from 800 to 102,400, and the geometric mean titer for severe and critical cases, moderate cases, asymptomatic and mild cases was 51,203, 20,912, and 9590 respectively.

**Conclusion:**

The development of a highly sensitive ELISA system for the detection of SARS-CoV-2 IgG antibodies is described herein. This system enabled a quantitative study of rSARS-CoV-2-N IgG antibody titers in COVID-19 patients, the occurrence of higher IgG antibody titers were found to be correlated with more severe cases.

**Supplementary Information:**

The online version contains supplementary material available at 10.1186/s12866-021-02401-0.

## Introduction

The 2019 novel coronavirus disease (named COVID-19 by the World Health Organization) represents a worldwide health challenge. The major clinical presentations include fever, fatigue, cough, breathlessness and pneumonia [1]. An associated novel coronavirus was identified from the throat swab sample of a patient, which was subsequently named severe acute respiratory syndrome coronavirus 2 (SARS-CoV-2) by the International Virus Classification Committee. Coronaviruses are single-stranded, positive-sense RNA viruses with an envelope, their genomes are the largest of any known RNA virus. SARS-CoV-2 is the third coronavirus that has been associated with human disease in the past two decades, all of which can cause fatal respiratory diseases [1, 2]. The other two viruses, SARS and MERS-CoV both caused large-scale outbreaks. As of July, 2003, 8096 SARS cases and 774 deaths in 29 countries were reported, with a total fatality rate of 9.6% [3]. MERS is still not under control, to date, it has caused 2494 confirmed cases and 858 deaths in 27 countries, with a case fatality rate of 34.4% [4]. Although the mortality rate of COVID-19 is not as high as SARS and MERS, it has become a major threat to global public health due to the large number of cases. A total of 125 million COVID-19 cases were reported to have occurred globally by the end of March 2021.

SARS-CoV-2 belongs to the *Betacoronavirus* genus lineage B, and genomic characterization of SARS-CoV-2 shows that it is closely related to bat SARS-like coronaviruses [5]. The diameter of the virus particles ranges from 60 to 140 nm, with unique spikes of about 8 to 12 nm in length [1]. The SARS-CoV-2 gene sequences include a 5′ untranslated region (UTR) and a 3′ UTR, between which the following genes are found: replicase complex (orf1ab), S, E, M and N, plus some uncertain open reading frames [1]. The SARS-CoV-2 S structural protein plays a major role in viral infection, it binds to the virions to the host cell receptor and participates in membrane fusion [6]. The E proteins play multiple roles in the viral replication cycle [7], virion release [8] and viral pathogenesis [9]. The M proteins play major role in regulation of the virosome assembly [10]. The N proteins enhance viral replication or translation of viral proteins, and package the viral RNA genome into new virions [11], hence the N proteins are considered a promising molecular target for effective drug treatments and vaccines [12, 13].

Laboratory confirmation is considered to be essential for the diagnosis of COVD-19 because the clinical manifestations are non-specific. Detection of viral RNA in respiratory tract samples is the gold standard for the diagnosis of COVID-19 currently. However, studies have shown that SARS-CoV-2 infects the lower respiratory tract and misdiagnosis can occur due to the need for high-purity samples, trained personnel, sophisticated sample processing equipment [14]. COVID-19 diagnosis is also complicated due to the presence of asymptomatic SARS-CoV-2 patients, and patients with a low viral load after the onset of the disease. Hence, methods for the detection of antibodies against SARS-CoV-2 proteins are urgently needed. Compared to PCR, serological testing is advantageous with a faster turn-around time, high throughput and is also less laborious [15]. Previous studies have shown that N proteins can play an important role in the immunological detection of virus specific antibodies due to their high immunogenicity [16–18].

Colloidal gold immunochromatography is currently the most commonly used antibody detection method in China, it is a simple and convenient method but it is a qualitative test that is not able to detect the antibody titer, it also has limitations regarding the sensitivity and specificity.

The study contained herein, utilized a recombinant SARS-CoV-2 nucleocapsid (rSARS-CoV-2-N) protein, which was expressed using an *Escherichia coli (E. coli)* system and purified under natural conditions. The rSARS-CoV-2-N protein was used to establish an IgG ELISA, which was compared with a colloid gold kit and a SARS-CoV-2 spike S1 protein IgG antibody ELISA kit since most of the ELISAs currently available are based on S protein using serial serum samples from COVID-19 patients (collected in Guizhou province) and healthy volunteers (collected before the SARS-CoV-2 outbreak). Moreover, a novel analysis was conducted using antibody titers from COVID-19 patients, which were cross referenced with their disease severity status during early convalescence. Further study of the relationship between the antibody titers of COVID-19 patients and the severity of their disease can improve the understanding of immune response after SARS-CoV-2 infection, provide information for the interpretation of epidemiology studies and help direct vaccine development.

## Results

### Expression and purification of rSARS-CoV-2-N

**Fig. 1:**
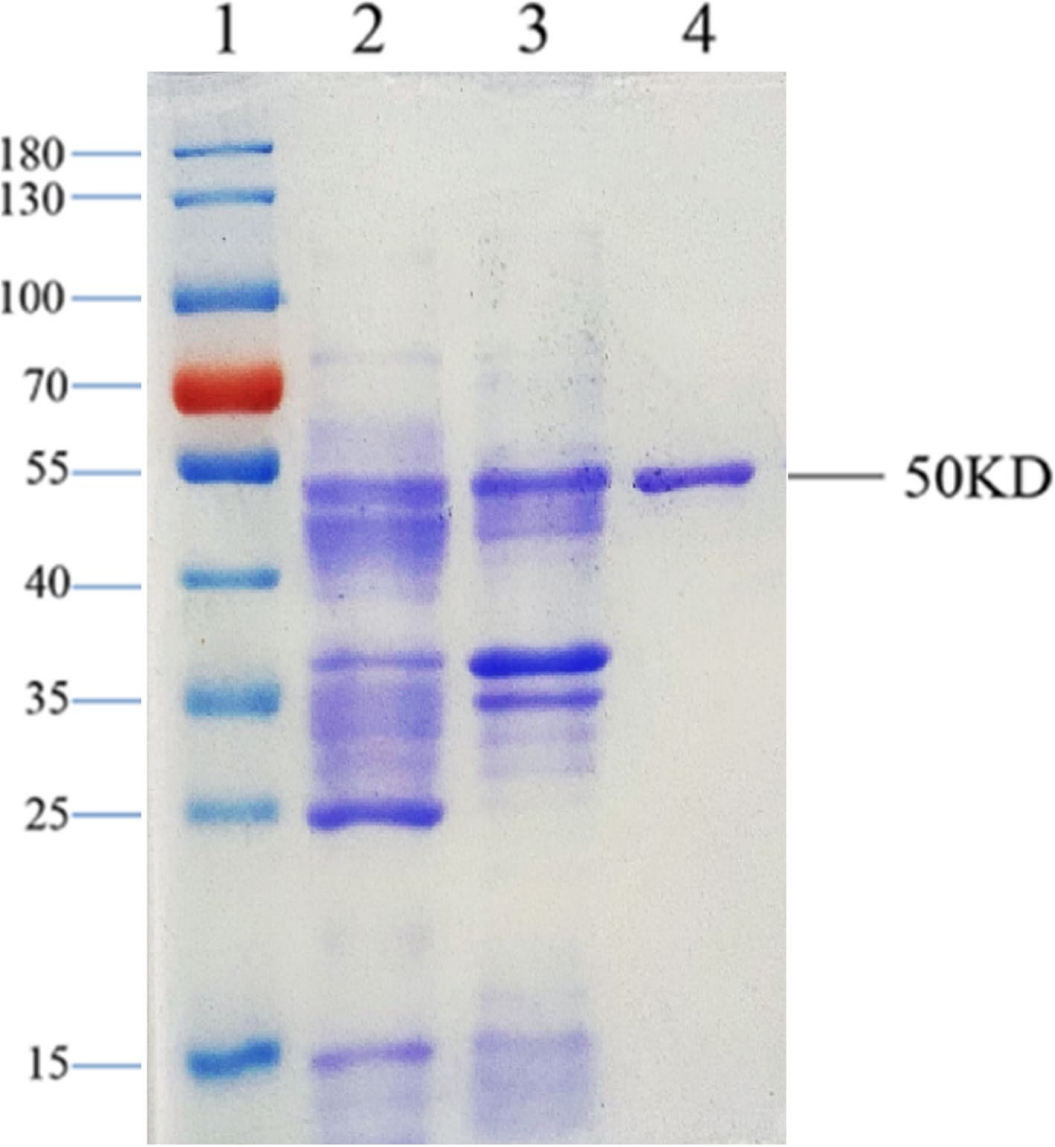
Expression and purification of rSARS-CoV-2-N protein. Recombinant plasmid containing the full length SARS-CoV-2 N gene was transformed into *E. coli* BL21 strain and induced with IPTG. E. coli cells were collected and dissolved in 10 mM PBS (PH 7.5) with 500 mM NaCl. After sonication, the *E. coli* cell lysate was centrifuged and the recombinant protein was purified from the supernatant by Talon™ IMAC affinity column. The *E. coli* cell lysate and the purified recombinant protein were analyzed using a 10% SDS-PAGE gel and stained with Coomassie brilliant blue staining. Lane 1: protein maker (Thermo Scientific); Lane 2: supernatant from sonicated *E. coli* cell lysate; Lane 3: pellet of sonicated *E. coli* cell lysate; Lane 4: purified recombinant protein

**Fig. 2:**
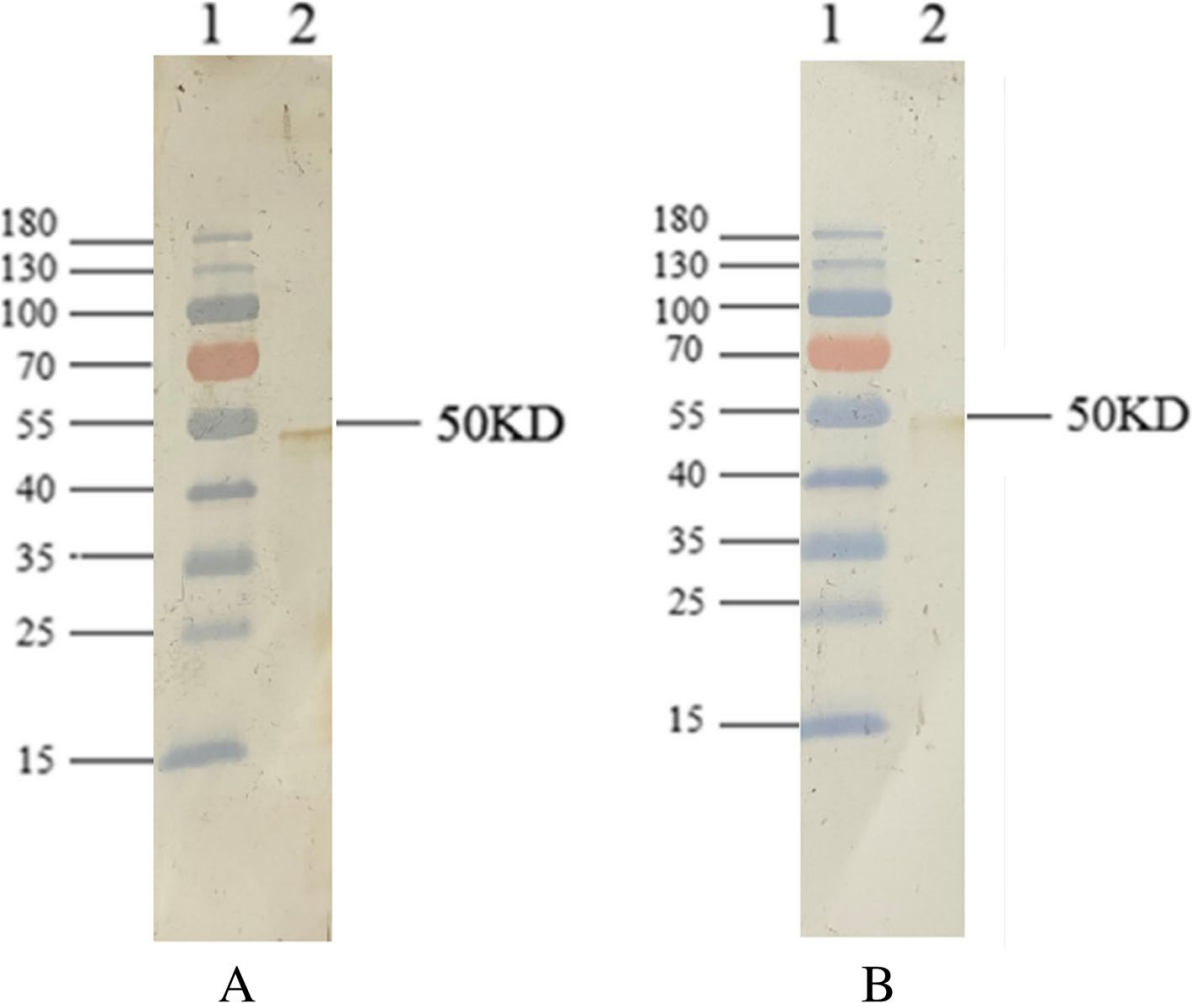
A western blot for the purified rSARS-CoV-2-N protein. The prestained protein marker and purified recombinant proteins were separated by SDS-PAGE and transferred to a PVDF membrane. Each membrane was incubated with diluted patient’s serum or mouse immune serum, followed by horseradish peroxidase conjugated-goat anti-human IgG or anti-mouse IgG (1:1000 dilution), and detected by DAB staining. (A) Reactivity of recombinant proteins to COVID-19 patient serum (1:400 dilution). Lane 1: protein marker; Lane 2: purified rSARS-CoV-2-N protein. (B) Reactivity of recombinant proteins to rSARS-CoV-2-N-immunized mouse serum (1:800 dilution). Lane 1: protein marker; Lane 2: purified rSARS-CoV-2-N protein

Full length *N* gene (nucleocapsid) encoding the full length nucleocapsid protein was amplified from a Japanese isolate and cloned into the pET28a (Beijing Solarbio Science & Technology, CN) expression vector. The sequence and reading frame of the N gene in the vector were confirmed via Sanger sequencing. The recombinant protein was successfully expressed in *E. coli* and purified by using a Talon metal affinity column under natural conditions. The analysis of the purified recombinant proteins was conducted using SDS-PAGE and Coomassie blue staining to reveal a single 50kD protein band as predicted by the nucleocapsid amino acid sequence (Fig. 1**,** lane 3). The identity of the rSARS-CoV-2-N protein was further confirmed via western blotting using with SARS-CoV-2-immunized mouse serum and COVID-19 patient serum (Fig. 2).

### Optimization of rSARS-CoV-2-N based IgG ELISA

To determine the amount of rSARS-CoV-2-N protein for coating and the dilutions of serum samples for the indirect IgG ELISA, checker board titration was performed by coating 2-fold serially diluted rSARS-CoV-2-N protein from 200 ng/well to 12.5 ng/well and serum 2-fold serially diluted from 1:100 to 1:800. The OD values (at 450 nm) of negative samples was lower and OD ratio of positive to negative samples was higher when using 50 ng/well for coating and a serum dilution of 1:400 (Table 1), both of which were chosen for later experiments.

**Table 1:** Checkerboard titration of rSARS-CoV-2-N based IgG ELISA

	200 ng/well	100 ng/well	50 ng/well	25 ng/well	12.5 ng/well
P 1:100	2.953	2.803	2.561	2.182	1.699
N 1:100	0.326	0.318	0.279	0.242	0.204
P/N	9.058	8.814	9.179	9.0165	8.328
P 1:200	2.627	2.411	2.022	1.597	1.34
N 1:200	0.28	0.264	0.259	0.236	0.173
P/N	9.382	9.133	7.807	6.767	7.746
P 1:400	2.095	1.899	1.592	1.051	0.874
N 1:400	0.254	0.196	0.129	0.106	0.04
P/N	8.248	9.689	12.341	9.915	21.85
P 1:800	1.228	1.038	0.797	0.686	0.567
N 1:800	0.215	0.193	0.17	0.084	0.032
P/N	5.712	5.378	4.688	8.167	17.719

The determination of optimal antigen coating concentration and dilution of serum

P**:** positive serum; N: negative serum; P/N: The ratio of the OD value of positive serum to negative serum

### Evaluation of recombinant SARS-CoV-2-N protein based indirect IgG ELISA

Among the 182 serum samples serially collected from 88 confirmed COVID-19 patients, 180 (98.9%) samples displayed a positive result using the rSARS-CoV-2-N protein-based indirect IgG ELISA and two were negative, 178 (97.8%) samples displayed a positive result using the SARS-CoV-2 spike S1 protein IgG antibody ELISA Kit (ABclonal, CN) and four were negative; whereas, 174 (95.6%) samples tested positive using a colloidal gold antibody detection kit (INNOVITA, CN) and eight samples were negative. Colloidal gold antibody detection kit, SARS-CoV-2 spike S1 protein IgG antibody ELISA kit and r-SARS-CoV-2-N based IgG ELISA correctly identified the healthy volunteers (180) serum samples as being negative. The OD value for the negative serum samples (the healthy volunteers serum samples collected before COVID-19 outbreak) ranged from 0.1–0.2, and from 0.4–3.9 for the positive serum samples (the COVID-19 patients collected in Guizhou province) in rSARS-CoV-2-N based IgG ELISA we established, and for the SARS-CoV-2 spike S1 protein IgG antibody ELISA kit, the OD value for the negative serum samples ranged from 0.01–0.04 and the positive serum samples ranged from 0.08–1.10. The sensitivity of our system was 100% with a specificity of 96.8% and had a 98.3% concordance with the colloidal gold antibody detection kit, furthermore, the sensitivity of our system was 100% with a specificity of 98.9% and had a 99.4% concordance with the SARS-CoV-2 spike S1 protein IgG antibody ELISA kit (Table 2**,** Table 3).

**Table 2:** Sensitivity and specificity of SARS-CoV-2-N-IgG ELISA with reference to the Colloidal gold antibody detection kit

	Colloidal gold antibody detection kit	r-SARS-CoV-2-N based IgG ELISA	Total
		Positive Negative	
Positive	174	0	174
Negative	6	182	188
Total	180	182	362
Concordance ^a^: 98.3%		Sensitivity ^b^: 100%	Specificity ^c^: 96.8%

χ^2^ = 3.702, *p* > 0.05

The sensitivity and specificity of the SARS-CoV-2-N IgG ELISA compared to the Colloidal gold antibody detection kit

a) (The number of positive samples detected by both methods + the number of samples determined to be negative by both methods)/the total number × 100

b) True positive (true positive + false negative) ×100

c) True negative (true negative – false positive) × 100

**Table 3:** Sensitivity and specificity of SARS-CoV-2-N-IgG ELISA with reference to the SARS-CoV-2 Spike S1 Protein IgG Antibody ELISA Kit

	SARS-CoV-2 Spike S1 Protein IgG antibody ELISA Kit	r-SARS-CoV-2-N based IgG ELISA	Total
		Positive Negative	
Positive	178	0	178
Negative	2	182	184
Total	180	182	362
Concordance ^a^: 99.4%		Sensitivity ^b^: 100%	Specificity ^c^: 98.9%

χ^2^ = 0.678, p > 0.05

The sensitivity and specificity of the SARS-CoV-2-N IgG ELISA compared to the SARS-CoV-2 spike S1 protein IgG antibody kit

a) (The number of positive samples detected by both methods + the number of samples determined to be negative by both methods)/the total number × 100

b) True positive (true positive + false negative) × 100

c) True negative (true negative – false positive) × 100

### Comparison of antibody titers for COVID-19 patients with different disease severity

**Fig. 3:**
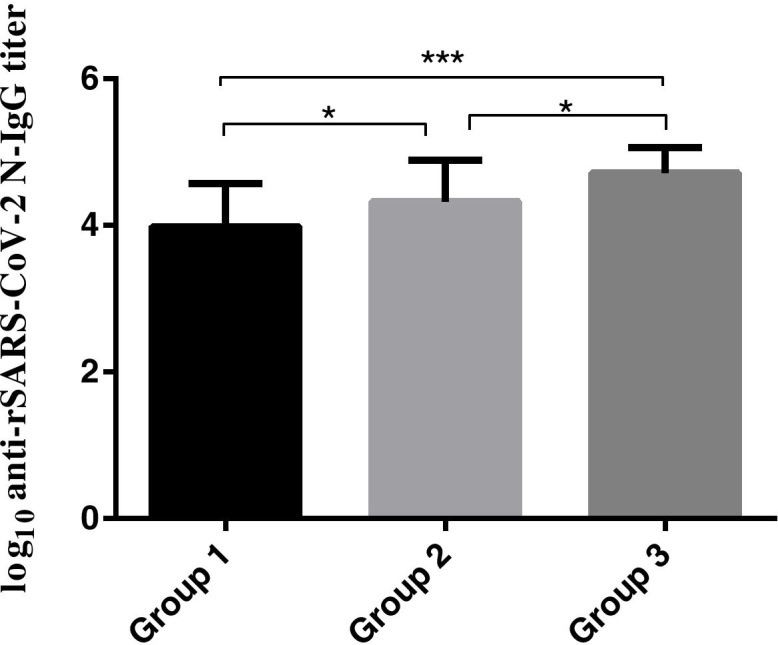
Difference in serum titer for the different severity. Group 1: SARS-CoV-2-N IgG antibody titer for 24 asymptomatic and mild cases; Group 2: SARS-CoV-2-N IgG antibody titer for 48 moderate cases; Group 3: SARS-CoV-2-N IgG antibody titer for 16 severe and critical cases. The significance of the difference was indicated using ‘* ‘(*p* < 0.05) and ‘*** ‘(*p* = 0.000)

To compare the immune response differences from COVID-19 patients with varying disease severity, 88 samples encompassing three different severity groups were checked for the IgG antibody titer. All the samples used for comparison were collected in the fifth week after the onset of the disease, which reflects the seroconversion in the patient. The first group included asymptomatic and mild cases (24 patients), for which the serum titer ranged from 800 to 51,200 with a geometric mean titer of 9590. The second group included patients with moderate cases (48 patients), for which the serum titer ranged from 800 to 102,400 with a geometric mean titer of 20,912. The third group were patients that experienced severe/critical disease (16 patients), the serum titer for the third group ranged from 12,800–102,400 with a geometric mean titer of 51,203. The antibody titer of third group was significantly higher than those of second group and first group (*p* < 0.05, and *p* = 0.000 respectively) (Table 4, Fig. 3).

**Table 4:** Geometric mean titer of different severity groups

Group	No. of serum samples	Range of antibody titer	Geometric mean titer
1	24	800–51,200	9590
2	48	800–102,400	20,912
3	16	12,800–102,400	51,203

The Geometric mean titer for the difference severity groups

Group 1 - asymptomatic and mild case; group 2 - moderate cases; group 3 - severe and critical cases

## Discussion

There are four main methods currently available for the detection of SARS-CoV-2 infection: 1) inoculation cultured cells with virus isolated from patient sample; 2) molecular detection techniques for viral nucleic acids, e.g. PCR; 3) serological testing; 4) antigen detection with specific monoclonal antibodies [19]. RT-PCR is the standard diagnostic method for SARS-CoV-2, but false-negative cases have been reported due to problems associated with sample collection and transportation, RNA extraction and enzyme inhibitors. Compared to RT-PCR assays, antibody detection assays are faster, do not require a high biosafety level laboratory and are generally less expensive. Previous studies have shown that virus-specific IgG levels allowed serologic diagnosis during the SARS epidemic, and for the SFTS virus and RVF virus [20–22]. IgG levels have been considered for the assessment of the serological response in COVID-19 patients and is beneficial for predicting the prognosis of patients [17, 18]. There are several reports detailing the use of recombinant SARS-CoV-2 nucleocapsid protein for the diagnosis of SARS-CoV-2 infection, which indicated that the recombinant nucleocapsid based ELISA had a high sensitivity for the detection in patient serum samples [23]. However, these studies use recombinant nucleocapsid protein as the antigen, the sample dilution ratio is relatively low, which consumes large volume of potentially precious samples and may cause nonspecific cross reactions. Kohmer et al. [24] evaluated six high-throughput detection methods for SARS-CoV-2 IgG antibodies, for the ELISA, the samples were diluted 1:100 in sample buffer; Nagasawa et al. [25] investigated three different ELISA test kits for anti-SARS-CoV-2 IgG antibodies in COVID-19 patients, with a sample dilution factor of 1:10; Xiang et al. [26] and Liu et al. [27] used an ELISA kit to detect serum SARS-CoV-2 antibodies with a sample dilution factor of 1:20 for IgG detection.

In the present study, rSARS-CoV-2-N protein was produced in *E.coli* and purified to near homogeneity using his-tag based affinity chromatography (Fig. 1), it was used as the assay antigen in the indirect IgG ELISA. The purified rSARS-CoV-2-N protein reacted with rSARS-CoV-2-N-immunized mouse serum and serum of a COVID-19 patient in a Western blotting assay (Fig. 2). Most of the expressed r-SARS-CoV-2-N protein was in a soluble form and the purification process was done under native conditions without the use of any detergent, thereby eliminating the need for a tedious refolding process that would be required for a denatured protein. Therefore, the production and purification procedures described in this study provide a simple and efficient way to obtain large quantities of pure rSARS-CoV-2-N.

The rSARS-CoV-2-N protein when used for the indirect IgG ELISA for serial serum samples from COVID-19 patients and healthy volunteers, showed that our in house rSARS-CoV-2-N protein-based indirect IgG ELISA had a 96.8% specificity and 100% sensitivity, with a 98.3% concordance to the INNOVITA colloidal gold kit, and had a 98.9% specificity and 100% sensitivity, with a 99.4% concordance when compared to the ABclonal SARS-CoV-2 spike S1 protein IgG antibody ELISA kit. Ultimately, the fact that the ELISA was able to detect the six patients that were missed by the INNOVITA colloidal gold kit, and two patients that were missed by the ABclonal SARS-CoV-2 spike S1 protein IgG antibody ELISA kit, demonstrated its usefulness for the sensitive and reliable clinical diagnosis of SARS-CoV-2 infection in humans using a relatively high dilution of 1:400.

The quantitative ELISA method was to compare the antibody titer of serum from patient’s with different COVID-19 severity (collected during the same period after the onset of infection), which demonstrated that the antibody titers were significantly different for the different severity groups. The geometric mean titer of the severe and critical patients was highest while asymptomatic and mild patients had the lowest mean (Table 4**)**. To our knowledge, this is the first report to quantitatively compare the antibody titer difference among COVID-19 patients with different severity. Wang et al. [28] reported that severely ill patients had more prolonged viral shedding in a variety of tissues than mildly ill patients. It is possible that patients with more severe COVID-19 have a higher viral load, which may trigger a stronger immune response, and cause a higher antibody titer than other cases. Further study of the relationship between the antibody titers of COVID-19 patients and the severity of their disease is needed to improve our understanding of the immune response after SARS-CoV-2 infection and shed light on epidemiology studies and vaccine development.

As our assay system only detects N-specific IgG antibodies. Therefore, the usefulness of the assay is more in epidemiological studies to detect seroconversions rather than for diagnosis of ongoing SARS-CoV-2 infections. Especially at early time points after infection when IgM antibodies are abundant which are not detected by our assay. Hence, the test result could be considered false negative early after infection due to absence of IgG antibodies.

## Conclusion

The rSARS-CoV-2-N protein based indirect IgG ELISA system presented herein is a safe diagnostic method that eliminated the use of infectious virus in the antigen production, which requires high level microbiological security facilities. The expression and purification procedures to produce r-SARS-CoV-2-N protein are a low cost scalable method that could be especially useful for large-scale epidemiological investigation. This protein can be used in an ELISA system that is more sensitive than the conventional colloidal gold kit (INNOVITA, CN) and the SARS-CoV-2 spike S1 protein IgG antibody ELISA Kit (ABclonal, CN) for detecting IgG antibodies in COVID-19 patients. Moreover, the ELISA is capable of quantitative detection at much higher serum dilutions (1:400 vs 1:10, 1:400 vs 1:100). This system enabled the observation that anti-SARS-CoV-2 nucleocapsid protein IgG antibody titers in patients with different COVID-19 severity after infection were significantly different, with higher antibody titers found in more severe cases.

## Methods

### Serum samples

Serum samples from 180 healthy volunteers collected before the SARS-CoV-2 infection outbreak at the Guizhou Provincial People’s Hospital. Additionally, 182 serial serum samples collected from 88 patients diagnosed with COVID-19 collected in Guizhou province. Of the COVID-19 patients, 42 were male and 46 were female. The age distribution of these patients ranged from 5 months to 84 years old. The patients had complications recorded including hypertension, diabetes, heart disease, surgery, chronic bronchitis, chronic gastritis, tuberculosis and hepatitis B virus infection.

According to diagnosis and treatment protocol for novel Coronavirus pneumonia (Trial Version 8) which was released by the National Health Commission & State Administration of Traditional Chinese Medicine, defined the clinical classification of asymptomatic COVID-19 patients as those who have no clinical symptoms, yet test positive for SARS-CoV-2 using respiratory tract and other specimens or have a positive IgM antibody test; mild cases were defined as those who had symptoms but no sign of pneumonia by chest X-ray imaging; moderate cases have fever and respiratory symptoms with radiological findings of pneumonia; severe cases and critical cases were typified by a severe respiratory response that require ICU care and failure of other organs .

### Expression and purification of the recombinant SARS-CoV-2-N protein

SARS-CoV-2 cDNA was prepared in BCL3 laboratory at the Nagasaki University, Japan. Briefly, SARS-CoV-2 virus TY-WK-521/20202 strain (Genebank accession number: LC522975) was isolated from a Japanese patient and propagated in a Vero cell line maintained at 37 °C in Eagle’s minimum essential medium supplemented with 2% fetal calf serum and 0.2 mM of each nonessential amino acid for four days. The viral RNA was extracted from the infected culture media and cDNA synthesized using a cDNA synthesize kit with random primers (Takara, JP). The full length SARS-CoV-2 N gene was amplified by RT-PCR using primers 5′- CAAGGATCCATGTCTGATAATGGACCCCAA-3′ (Bam HI site underlined) and 5′- TGCGTCGACTTAGGCCTGAGTTGAGTC-3′ (Sal I site underlined). The PCR product was successfully cloned into the BamHI and Sall sites of the pET28a expression vector. The sequence and reading frame of the N gene within the recombinant plasmid was confirmed by DNA sequencing. The rSARS-CoV-2-N protein was produced by inserting the recombinant plasmid into *E. coil* (strain *BL-21*), which was cultured at 37 °C in Luria-Bertani (LB) medium containing 50 μg/ml of kanamycin. When the optical density at 600 nm (OD_600_) of the culture reached 0.5, the expression of the recombinant proteins was induced by the addition of 0.2 mM isopropyl-β-D-thiogalactopyranoside (IPTG) for 5 h. The cells were harvested by centrifugation, washed in phosphate-buffered saline (PBS) solution, and resuspended in 10 mM PBS PH 7.5 with 500 mM NaCl and frozen at − 80 °C. After freezing and thawing three times, the cell suspension was sonicated for 10 min with an interval of 3 s between pulses and centrifuged at 13,000 g for 30 min at 4 °C. The supernatant was passed through a Talon™ IMAC resin column (Clontech, US). After being washed with binding buffer (10 mM PBS with 500 mM NaCl containing 30 mM imidazole, PH 7.5), the purified protein was eluted using elution buffer (10 mM PBS with 500 mM NaCl containing 250 mM imidazole, PH 7.5). The protein solution was aliquoted and stored at a final concentration of 10% glycerol at − 80 °C until use. The protein concentrations were determined by the Bradford method using a Bio-Rad protein assay reagent kit (Bio-Rad, USA), and the purity of the protein was analyzed by sodium dodecyl sulfatepolyacrylamide gel electrophoresis (SDS-PAGE).

### Western blot analysis

A western blot analysis was done as mentioned [27]. Briefly, the proteins were separated in a 10% polyacrylamide gel were transferred to a polyvinylidene difluoride (PVDF) membrane (Sigma-; US) using a semidry electroblotter (Sartorius, DE). The membrane was blocked with PBS-T with 5% skimmed milk (BD, US) overnight at 4 °C to prevent nonspecific staining. After which, the membrane was exposed to reaction with rSARS-CoV-2-N-immunized mouse serum (1:800 dilution), or COVID-19 patient serum (1:400 dilution) for 1 h at 37 °C; and subsequently incubated with horseradish peroxidase conjugated-goat anti-mouse IgG, or anti-human IgG (1:1000 dilution) for 1 h at 37 °C. The reaction was visualized by dimethyl amino benzidine (DAB) staining.

### Checkerboard titration of rSARS-CoV-2-N based IgG ELISA

To evaluate the usefulness for diagnosis of the rSARS-CoV-2-N protein, an indirect IgG ELISA was established for the laboratory diagnosis of SARS-CoV-2 infection in human’s serum sample. The rSARS-CoV-2-N protein was used as antigen. The optimal concentrations of recombinant nucleocapsid protein were determined by checkerboard titration with different dilutions of coating recombinant protein. Briefly, purified rSARS-CoV-2-N protein was diluted in a 2-fold serial dilution started from 200 ng/well/100 μl to 12.5 ng/well/100 μl in phosphate buffered saline (PBS) and coated to ELISA plate overnight at 4 °C respectively. After washing the plate, 2 positive and 2 negative serum samples were diluted in a 2-fold serial dilution started from 1:100 to 1:800 and 100 μl of the diluted sera was reacted with the coated rSARS-CoV-2-N protein wells at 37 °C for 1 h respectively. After washing the plate, 100 μl of 1:30,000 diluted horseradish peroxidase-conjugated goat anti-human IgG was added to each well and reacted at 37 °C for 1 h. After washing the plate, the color was developed by TMB substrate and the OD value of each well recorded.

### ELISA procedures using the recombinant nucleocapsid protein

To evaluate the diagnostic utility of the rSARS-CoV-2-N protein as an antigen in an indirect IgG ELISA to detect SARS-CoV-2 infection in human serum sample. The optimal concentrations of recombinant nucleocapsid protein were determined by checkerboard titration with different dilutions of recombinant protein coating. Ninety-six-well Nunc immunoplates (Nest Biotechnology, CN) were coated with 50 ng recombinant nucleocapsid protein antigen per ELISA well in 100 μl phosphate buffered saline (PBS) overnight at 4 °C. After the immunoplates were washed three times with PBS-Tween 20, 100 μl of 1:400 human serum diluted in PBS-Tween 20 with 5% nonfat milk (BD, US) was add to each well and incubated for 1 h at 37 °C. The plates were washed six times with PBS-Tween 20 and incubated for 1 h at 37 °C with 100 μl 1:30,000 horseradish peroxidase-conjugated goat anti-human IgG (American Qualex, US). After washing six times with PBS-Tween 20, 100 μl of TMB single-component substrate solution (Beijing Solarbio Science & Technology, CN) was added to each well and incubated in the dark at room temperature for 30 min. The reaction was stopped by adding 100 μl HCI (1 mol/L) to each well. OD values at 450 nm were recorded on a continuous wavelength ELISA reading instrument (Epoch). Each serum sample was tested in duplicate, and the mean OD for each sample was calculated. Any OD more than twice the mean OD of the negative control serum was considered positive.

Serum samples collected at fifth week after the onset of the disease were used to compare the IgG antibody difference of the COVID-19 patients with different severity. ELISA titers for these sera were calculated from standardized reciprocal dilution values using Thermo-Labsystem’s Ascent photospectrometric data analysis software, version 2.6 according to an established protocol [20].

### Detection of SARS-CoV-2 IgG antibody by colloidal gold kit

The detection of serum IgG antibodies for SARS-CoV-2 using the colloidal gold kit (INNOVITA, CN) which used colloid gold labeled SARS-CoV-2 N and S fusion protein as an indicator was conducted according to the manufacturer’s instructions. Briefly, 10 μl of serum was added to the sample hole, 2 drops of serum sample diluent was added immediately, the result was observed within 15 min. If a clear purple-red band appeared on the T line and C line of the interpretation window, it is judged as the IgG antibody positive. If only the C line has the purple-red band, it is judged as the IgG antibody negative.

### Detection of SARS-CoV-2 IgG antibody by SARS-CoV-2 spike S1 protein ELISA kit

The detection of serum IgG antibodies for SARS-CoV-2 using the SARS-CoV-2 spike S1 protein IgG antibody ELISA Kit (ABclonal, CN) was done according to the manufacturer’s instructions. Briefly, 100 μl of control antibody and 1:100 serum samples diluted in dilution buffer was add to each well of the 96 well plate coated with SARS-CoV-2 spike S1 protein and incubated for 2 h at 37 °C, then the plates was washed three times with wash buffer, 100 μl of secondary antibody working solution was added to each well, and the plates were incubated at 37 °C for 1 h. After three times washing with wash buffer, 100 μl substrate solution was added to each well and incubated in the dark at 37 °C for 15–20 min. The reaction was then stopped by adding 50 μl of stop solution. Finally, the OD values at 450 nm were recorded on a continuous wavelength ELISA reading instrument (Epoch) within 5 min. OD value more than twice the negative control was considered positive.

### Statistical analysis

The data was presented using the mean of the original data after logarithmic conversion. The significance of the differences between the groups was determined using a one-way ANOVA, the differences between the N-based IgG ELISA and colloid gold antibody detection kit, the differences between the N-based IgG ELISA and S1 protein IgG ELISA kit were determined by the McNemar square test, with SPSS version 22.0.

## Supplementary Information

**Additional file 1.**

## Data Availability

The DNA and protein data analysed during the current study are available in the Genebank (accession number: LC522975). Other data generated in this study have been included within this article.

## Abbreviations

DAB: Dimethyl amino benzidine
*E. coli*: *Escherichia coli*
ELISA: Enzyme linked immunosorbent assay
ICF: infected culture fluid
IgG: Immunoglobulin G
IgM: Immunoglobulin M
OD: Optical density
PBS-T: 0.01 M PBS with 0.1% (vol/vol) Tween 20
rSARS-CoV-2-N: recombinant SARS-CoV-2-N.
