# Supplementary material for: IgG antibody titers against SARS-CoV-2 nucleocapsid protein correlate with the severity of COVID-19 patients
Source: BMC Microbiol. 2021 Dec 18;21:351. doi: 10.1186/s12866-021-02401-0 (PMC8683808; doi:10.1186/s12866-021-02401-0)
Supplement: Supplementary file 1 — Additional file 1. [file 12866_2021_2401_MOESM1_ESM.docx]

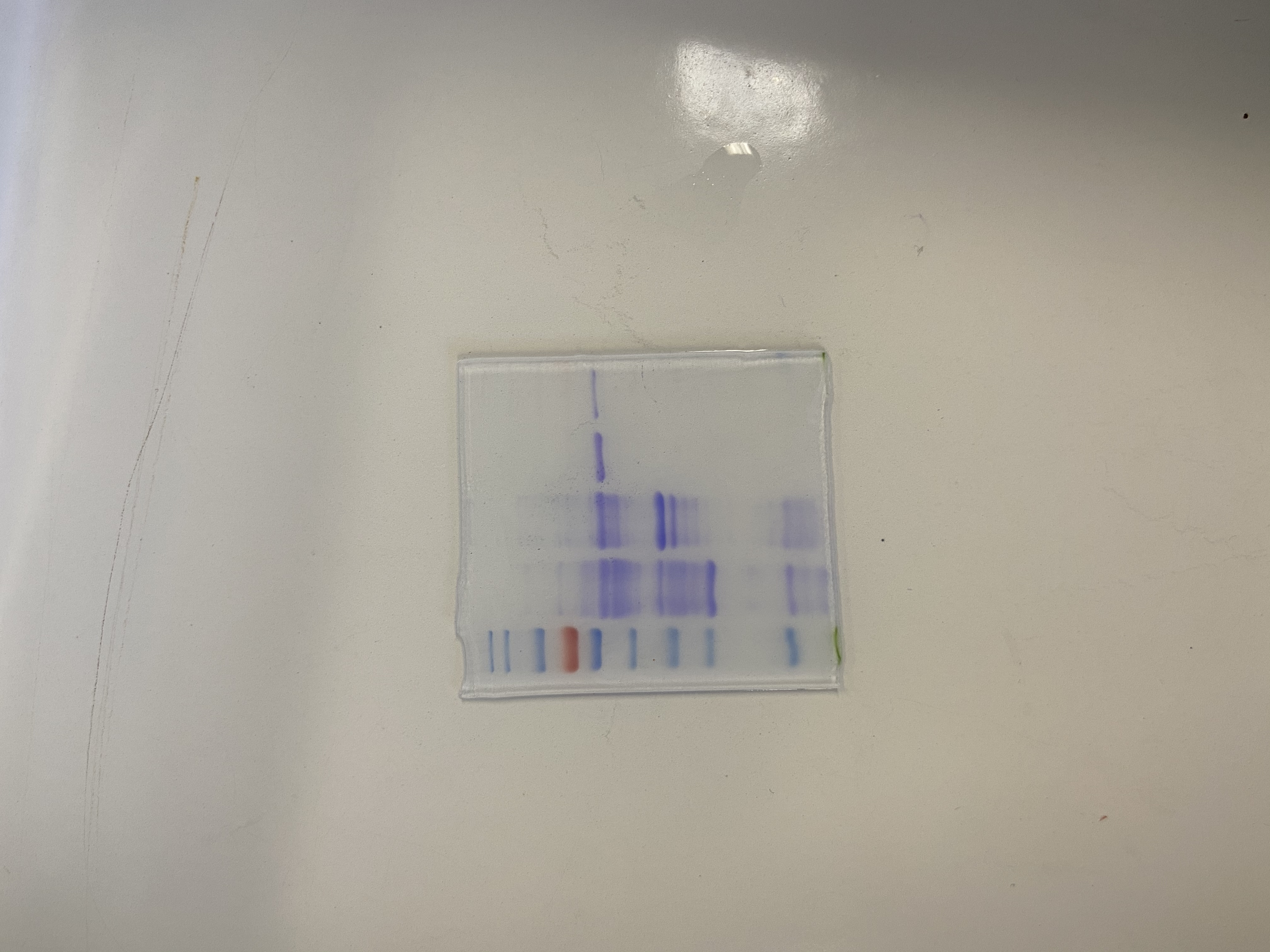


**FIG. 1. Expression and purification of rSARS-CoV-2-N protein.**

Recombinant plasmid containing the full length SARS-CoV-2 N gene was transformed into E. coli BL21 strain and induced with IPTG. E. coli cells were collected and dissolved in 10 mM PBS (PH 7.5) with 500 mM NaCl. After sonication, the E. coli cell lysate was centrifuged and the recombinant protein was purified from the supernatant by Talon^TM^ IMAC affinity column. The E. coli cell lysate and the purified recombinant protein were analyzed using a 10% SDS-PAGE gel and stained with Coomassie brilliant blue staining. Lane 1: protein maker (Thermo Scientific); Lane 2: supernatant from sonicated E. coli cell lysate; Lane 3: pellet of sonicated E. coli cell lysate; Lane 4: purified recombinant protein.


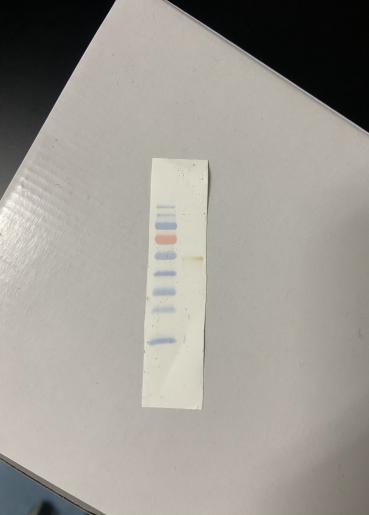

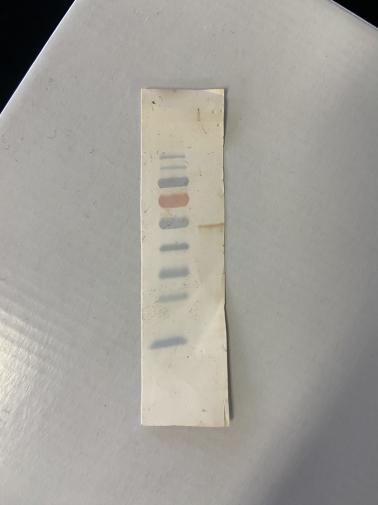


**A B**

**FIG. 2. A western blot for the purified rSARS-CoV-2-N protein.**

The prestained protein marker and purified recombinant proteins were separated by SDS-PAGE and transferred to a PVDF membrane. Each membrane was incubated with diluted patient’s serum or mouse immune serum, followed by horseradish peroxidase conjugated-goat anti-human IgG or anti-mouse IgG (1:1000 dilution), and detected by DAB staning. (A) Reactivity of recombinant proteins to COVID-19 patient serum (1:400 dilution). Lane 1: protein marker; Lane 2: purified rSARS-CoV-2-N protein. (B) Reactivity of recombinant proteins to rSARS-CoV-2-N-immunized mouse serum (1:800 dilution). Lane 1: protein marker; Lane 2: purified rSARS-CoV-2-N protein.
